# The Impacts of Farming Activities on the Coevolutionary Structure of Plant Rhizosphere Soil Microbial Communities

**DOI:** 10.3390/microorganisms13061216

**Published:** 2025-05-26

**Authors:** Qiuju Wang, Yu Jiang, Gang Mi, Xin Liu, Jiahe Zou, Jingyang Li, Zhenhua Guo

**Affiliations:** 1 Heilongjiang Provincial Key Laboratory of Soil Environment and Plant Nutrition, Heilongjiang Academy of Agricultural Sciences, Harbin 150086, China; 2 National Soil Quality Aihui Observation Experimental Station, Heihe Branch of Heilongjiang Academy of Agricultural Sciences, Heihe 164300, China; 3 Heilongjiang Academy of Agricultural Sciences, Animal Husbandry Research Institute, Harbin 150086, China

**Keywords:** Cyanobacteriales, *Fusarium*, plants, *Rhizobia*, soil peroxidase

## Abstract

Human agricultural activities can impact the soil microbial ecosystem, but the future implications of such changes remain largely unknown. This study aimed to explore how soil microbes survive and reproduce under the pressure of human agricultural cultivation and whether they resist or adapt. A 10-year continuous experiment was conducted, planting a maize and soybean rotation (control group), alfalfa (legume), and wheat (poaceae) to study the impact of different crop planting on soil microbial communities. During the experiment, the physical and chemical properties of the soil samples were measured, and the rhizosphere microbial communities were analyzed. Different crop plantings had varying effects on soil microbial species diversity, but these differences were relatively limited. The relative abundance of Cyanobacteriales (order) was higher in wheat than in alfalfa. Moreover, Cyanobacteriales were positively correlated with soil peroxidase, thereby promoting wheat growth. In addition, nutrition for fungi is mainly derived from decaying straw and plant roots. This study divided soil microbes under agricultural cultivation conditions into three categories: adaptive microbes, neutral microbes, and resistant microbes. At the ecological level of plant rhizosphere microbes, the plant rhizosphere soil microbial community showed a coevolutionary relationship with human cultivation activities. Future research needs to pay more attention to the adaptability of soil microbial communities to agricultural cultivation and the potential impact of this adaptability on the global ecosystem.

## 1. Introduction

To meet the basic needs for survival, humanity has historically converted forests and grasslands into farmland for crop cultivation [1]. This shift in land use patterns has altered the carbon/nitrogen cycles of the biosphere and the structure and function of soil microbial communities [2]. Faced with the increasing pressure of population growth, the area of arable land is struggling to keep pace [3]. This has prompted us to seek higher grain output on limited arable land [4]. Through the use of chemical fertilizers and refined field management, grain production has increased to meet human food demands [5]. However, the use of chemical fertilizers has further changed the composition of plant rhizosphere soil microbial communities, leading to a significant increase in microbial populations adapted to agricultural cultivation. The ecological effects and long-term impacts on biogeochemical processes are not yet fully understood.

Zhu (2024) proposed a new concept, the rhizosphere life community (Rhizobiont), which explains the relationships between plants, roots, the rhizosphere, the hypersphere, and soil microorganisms [6]. The joint action of plants and rhizosphere microbial communities can fix atmospheric carbon and nitrogen in the soil, playing a key role in the carbon/nitrogen cycle of the biosphere [7]. *Rhizobia* (*Mesorhizobium*) fix atmospheric nitrogen to provide nutrients for plant growth or further mineralization [8,9,10,11]. At the same time, plants return carbon sources and other nutrients to *Rhizobia* [12]. In addition, *RB41*, *Bradyrhizobium*, and *Streptomyces* play important roles in the carbon fixation process, generating about 50% of the carbon in the soil [7].

Traditional research considers that the microbial communities in the plant rhizosphere can be distinguished into beneficial and harmful microorganisms based on their interactions with plants [13]. This study focuses on the beneficial microorganisms *Rhizobia* and *Saccharomyces*. *Rhizobia* are widely recognized as beneficial bacteria [9,10,11,14], fixing atmospheric nitrogen and meeting the needs for plant growth [8,9,10,11]. It is generally believed that soil peroxidase is closely related to plant disease and stress resistance [14,15]. Studies have found that *Saccharomyces* is positively correlated with soil peroxidase [16,17]. The harmful microorganism discussed in this study is *Fusarium*, which is a major pathogenic fungus that causes plant diseases [18,19]. The species of *RB41* discussed in this study is neither a beneficial nor a harmful microorganism. *RB41* can absorb carbon sources and fix them in the soil [7].

To explore how soil microbial communities respond to human agricultural cultivation, this study poses a key scientific question: how do these microorganisms maintain their survival and reproduction, by resisting or adapting? To answer this question, we propose a hypothesis defining soil microbes under agricultural cultivation conditions into three categories: adaptive microbes, neutral microbes, and resistant microbes. Through 10 years of continuous field experiments, we observed and recorded the coevolutionary process of plant rhizosphere soil microbial community structures under cultivation conditions. This long-term study not only reveals how soil microbial communities interact with plants but also has positive significance in evaluating the impact of chemical fertilizer use and refined field management on plant rhizosphere soil microbial communities, providing important references for sustainable agricultural development.

## 2. Materials and Methods

### 2.1. Experimental Area and Location

**Figure 1:**
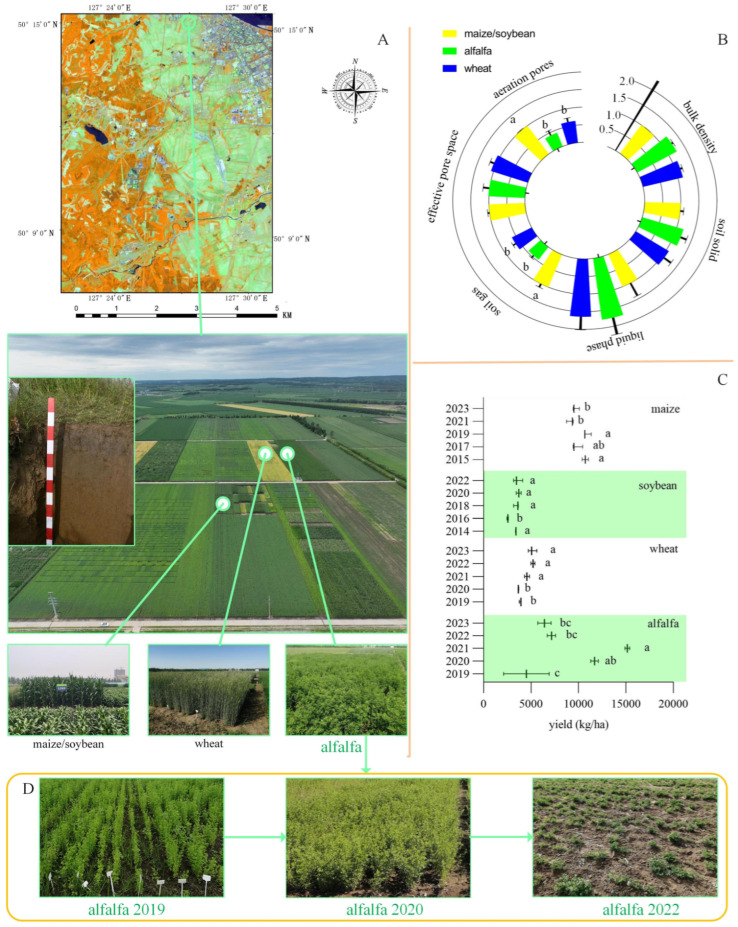
Location of the experiment, crop yields, and soil physical properties. (**A**) Location of the experiment. The satellite remote sensing map shows that the experimental site is located in Heihe City, Heilongjiang Province, and the soil profile morphology indicates that the soil type is dark brown soil. The planting sites for wheat (poaceae), alfalfa (legume), and maize and soybean rotation (control) were very close to each other and were planted and managed by professional experimental personnel. (**B**) Soil physical properties. The data were normalized, with the data for maize defined as 1, and the bars in this figure represent the standard error (SE). Different letters indicate significant differences. (**C**) Yield data from 2014 to 2023. The yield of alfalfa reached the maximum value in 2022 and 2023, then significantly decreased. The bars in this figure represent the maximum and minimum values. Different letters indicate significant differences. (**D**) Photos of alfalfa growth. planted in 2019, the growth condition was good in 2020, and some plants died in the spring of 2022.

The experimental farmland was located within the experimental fields of the Heihe Branch of the Heilongjiang Academy of Agricultural Sciences (Heihe city, Heilongjiang, China; Figure 1A) at 50°15′ N and 127°27′ E, with an altitude of 160 m above sea level. According to the Genetic Soil Classification of China (GSCC) [20], the soil type is dark brown soil. The climate is characterized as a temperate continental monsoon climate, with an average annual temperature ranging from −2.0 to 1.0 °C, a frost-free period of 105–120 days, an average annual precipitation of 450–510 mm, and an average annual evaporation of 650 mm. From May to September, the temperature is relatively high, with large diurnal temperature differences, and precipitation during the crop growth period accounts for 75% of the annual total, with ample sunlight. The satellite remote sensing map of the experimental site was annotated with latitude and longitude using Arc GIS (ESRI Arc MapTM 10.8).

### 2.2. Experimental Design and Sample Collection

A 10-year continuous experiment was performed to explore the long-term effects of different crop rotation systems on soil physical and chemical properties and microbial community structures. The experiment was divided into two stages. In the initial 5 years (2014–2018), a maize and soybean rotation was used to homogenize the soil physicochemical properties and microbial communities. In the subsequent 5 years (2019–2023), the land was divided into three treatment groups, with the maize and soybean rotation (control) continuing as the control group, and new groups for alfalfa (legume) and wheat (poaceae) were added. In all treatment groups, crop straw was managed by in situ return to the field to simulate natural material cycling. In terms of chemical fertilizer application, we conducted standardized management according to the recommended amounts listed in Table 1. In the 10-year experiment, we continuously collected and recorded crop yield data and performed statistical analysis using GraphPad Prism (version 9.3.1). To further study the microbial communities in plant rhizosphere soil, samples of plant rhizosphere soil were collected from the 10–15 cm soil layer on 27 September 2023. The five-point sampling method proposed by [21] was used for sample collection. Soil samples for physicochemical analysis were collected in special sample collection bags, and samples for microbial analysis were quickly placed in centrifuge tubes and preserved in liquid nitrogen after collection.

### 2.3. Determination of Soil Physical and Chemical Properties

After transporting the rhizosphere soil samples to the laboratory, further processing and analysis were carried out. The pretreatment referred to the “NY/T 1121.1-2006” standard [22], which involved removing plant residue, intrusive bodies (stones), and new formations (iron and manganese concretions) from the soil, after which the samples were air-dried, crushed with a mortar, and sifted through 2 mm and 0.25 mm screens to collect uniform samples suitable for experimental analysis. We measured the physical properties of the soil, namely, the soil aeration pores, effective pore space, soil liquid phase, soil gas, soil solid, and soil bulk density [23]. The physical properties of the soil in the control group were defined as the standard value 1, and after normalization, we effectively compared the physical properties of the experimental group’s soil with the control group, thereby assessing the impact of different crop planting patterns on the soil physical structure. For example, the soil bulk density was calculated as follows:

$$soil\;bulk\;density(wheat)=100\times \left(\frac{wheat\;soil\;bulk\;density}{maize\;soil\;bulk\;density}\right)\%$$

In this study, we conducted a comprehensive analysis of the chemical properties of the rhizosphere soil samples, and the chemical indicators tested were sucrose enzyme, catalase, urease, peroxidase, available phosphorus, available potassium, total potassium, total organic carbon, total nitrogen, total phosphorus, biomass carbon, biomass nitrogen, biomass phosphorus, total organic carbon, and pH values. The experimental methods were performed following [21]. The specific detection methods were conducted according to “NY/T 1121.24-2012” [24]. The data obtained were statistically analyzed using one-way analysis of variance (ANOVA) and completed using SPSS Statistics (version 17.0).

### 2.4. Soil Microbial Measurement

The extraction and sequencing of microbial DNA from rhizosphere soil samples were carried out by Biomarker Technologies Co., Ltd. (Beijing, China). Briefly, total DNA was extracted using a TGuide S96 Magnetic Soil DNA Kit (Tiangen Biotech Co., Ltd., Beijing, China). Then, the hypervariable region V3–V4 of the bacterial 16S rRNA gene and ITS of the fungal gene were detected.

#### 2.4.1. Species Taxonomic Analysis

In this study, soil samples under wheat (poaceae), alfalfa (legume), and maize and soybean rotation (control) planting conditions were grouped. According to the number of operational taxonomic units (OTUs), we selected the top 80 characteristic sequences with the highest abundance ratio in each crop group. The QIIME2 software (version 2023.2; https://qiime2.org) was used for multiple sequence alignment, and a phylogenetic tree was constructed. Combined with the phylogenetic tree and species classification abundance data, data visualization was achieved using the R package ggtreeExtra, which was displayed in the form of a species circular phylogenetic tree. In addition, to explore the differences in the composition of soil microbial communities under wheat (poaceae), alfalfa (legume), and maize and soybean rotation (control) planting conditions, a significant difference analysis of all OTUs was conducted, which was carried out through BMKCloud (www.biocloud.net), and the results were displayed in a ternary phase diagram.

#### 2.4.2. Species Diversity Analysis

Based on the grouping of wheat (poaceae), alfalfa (legume), and the maize and soybean rotation (control), we studied the species diversity of microorganisms in the soil under different crop planting conditions. Principal component analysis was used to reveal the differences. The analysis was performed using GraphPad Prism (version 9.3.1). For the similarity of microbial communities, we used analysis of similarities (Anosim), a statistical method for multidimensional data group similarity. Anosim was performed using the vegan package in the R language, and the results were visualized using Python plotting (version 3.10; https://www.python.org). Furthermore, to better understand the compositional differences of soil microbial communities and their relevance to crop planting patterns, we employed Partial Least Squares Discrimination Analysis (PLSDA) using MetaboAnalyst 6.0 (https://www.metaboanalyst.ca/).

#### 2.4.3. Plant and Rhizosphere Microbial Coevolution

In this study, based on the grouping of wheat (poaceae), alfalfa (legume), and the control (maize and soybean rotation), microbial community analysis was conducted using the BMKCloud (www.biocloud.net) platform. Special attention was given to several key microbial groups, including *Rhizobia*, Cyanobacteriales (order), *RB41*, and fungi (*Saccharomyces*). These microbial groups were plotted as species composition circular diagrams at the genus level. To further understand the functional potential of these microbial communities, the Phylogenetic Investigation of Communities by Reconstruction of Unobserved States (PICRUSt2; version 2.2.3; https://huttenhower.sph.harvard.edu/picrust/) software was used to predict functional genes for all detected effective OTUs.

### 2.5. Environmental Factors and Microbial Correlation Analysis

To fully assess the impact of crop planting on the structure of soil microbial communities, we conducted a comprehensive analysis of all data from the wheat (poaceae), alfalfa (legume), and control (maize and soybean rotation) groups. During the research process, we listed peroxidase, total potassium, and available phosphorus as environmental factors for correlation analysis at the genus level of rhizosphere microbes. We first conducted a Pearson correlation analysis between species diversity and environmental factors, aiming to explore the correlation characteristics between species (bacteria/fungi) and environmental factors. Through a Mantel analysis, a heatmap and network combination diagram were drawn, with the correlation threshold set to 0.3, the correlation *p*-value threshold set to 0.05, and the number of nodes in the network analysis set to 80, with 100 edges.

### 2.6. Peroxidase Protein 3D Structure Docking

In this study, we initially downloaded the glutathione peroxidase gene sequence (C4S55_1268) of *Saccharomyces* from the Ensembl Fungi database, which comprised 504 base pairs encoding 167 amino acids, on 17 January 2024. Concurrently, we also retrieved the glutathione peroxidase gene sequence (Gene ID 123097394) of wheat from the NCBI database on the same date. Utilizing the Swissmodel tool (accessible at https://swissmodel.expasy.org/, last accessed on 17 January 2024), we constructed the high-level structures of peroxidase for both *Saccharomyces* and wheat. Subsequently, we obtained the ligand structure of L-glutathione (CID 124886) from the PubChem database (accessed on 17 January 2024). Referring to our previously described methodology [25], we conducted a docking experiment between the protein and the ligand. The AutoDock Vina software version 4.2.6 (available at https://vina.scripps.edu/) was employed to perform protein and ligand docking, with 100 iterations used to evaluate the affinity between the protein and the ligand. The docking results were analyzed and visualized using PyMOL version 2.6.0 (accessible at https://www.pymol.org/).

## 3. Results

### 3.1. Changes in Soil Physical Properties and Crop Yields

Figure 1B describes the changes in soil physical properties after five years of cultivation. There were no significant differences in the effective pore space, soil liquid phase, soil solid, or soil bulk density among the three crops. However, the soil gas and soil aeration pores in the maize and soybean rotation (control) were significantly higher than those in wheat (poaceae) and alfalfa (legume). Figure 1C depicts the fluctuations in the crop yields over the 10 years. The wheat yield in the last 3 years (2021–2023) of the study was significantly higher than in the first 2 years (2019 and 2020). In contrast, the alfalfa yield in 2022 and 2023 was significantly lower than that in 2021 (Figure 1C,D). The alfalfa planted in 2019 showed good growth in the following 2 years (2020 and 2021), but some plants began to die in the spring of 2022, leading to a direct reduction in yield for that year and the following year.

### 3.2. Coevolutionary Structure of Plant Rhizosphere Soil Microbial Communities

#### 3.2.1. Taxonomic Analysis of Species

**Figure 2:**
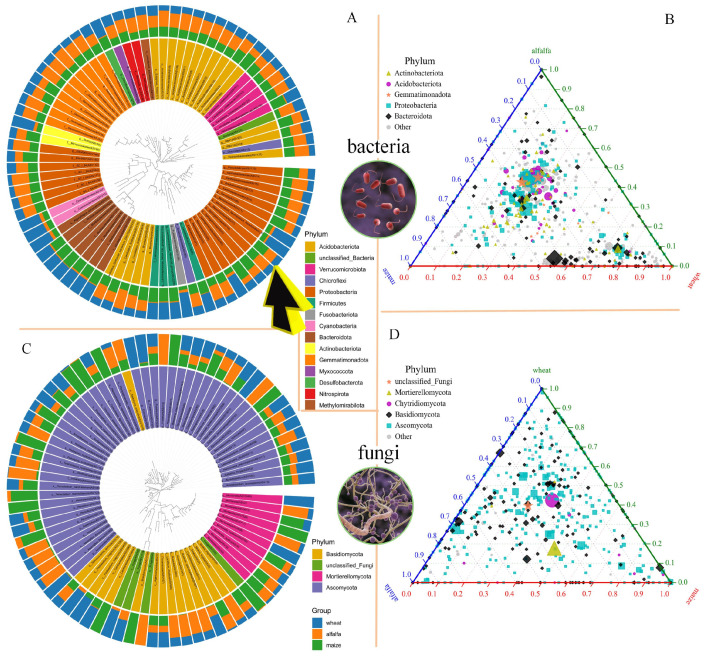
Sample community distribution of species phylogenetic tree and ternary phase diagram of group difference analysis. (**A**) Circular phylogenetic tree of rhizosphere soil bacteria; 15 phyla were detected, with four genera dominant in wheat. (**B**) Ternary phase diagram of the difference in rhizosphere soil bacteria among the wheat (poaceae), alfalfa (legume), and maize and soybean rotation (control) groups. A total of five phyla were detected. (**C**) Circular phylogenetic tree of rhizosphere soil fungi. A total of four phyla were detected, with genera *Echria* (ASV5585) and *Mortierella* (ASV5584) only detected in wheat. Genus *Mortierella* (ASV7326) was only detected in the maize and soybean rotation (control). In this figure, c_ represents Class, o_ represents Order, f_ represents Family, and g_ represents Genus. (**D**) Ternary phase diagram of the difference in rhizosphere soil fungi among wheat, alfalfa, and maize groups. The three corners of the triangle represent wheat, alfalfa, and maize. The three sides were used to measure the species abundance of the corresponding colored samples. The circles (or squares) in the triangular diagram represent all the species classified at a certain taxonomic rank, and the size of the circles (or squares) represents the average relative abundance of the species.

In this study, a total of 14,424 bacterial OTUs and 7578 fungal OTUs were detected. Figure 2A describes the circular phylogenetic tree of rhizosphere soil bacteria, with a total of 15 phyla detected and 4 genera dominant in wheat (poaceae). *Marivivens*, *Rothia*, *Fusobacterium*, and *Methylocystis* were significantly more abundant in the rhizosphere of wheat (poaceae) than in that of the control (maize and soybean rotation) and alfalfa (legume). Notably, *Rhizobia* (*Mesorhizobium*) indicated by the arrow, was less abundant in the rhizosphere of wheat than in maize and alfalfa. Additionally, Cyanobacteriales, indicated in light pink, was more abundant in the rhizosphere of wheat than in maize and alfalfa. These findings are further analyzed in the subsequent discussion. Figure 2C describes the circular phylogenetic tree of rhizosphere soil fungi, with the top 80 fungi belonging to 4 phyla. Figure 2B,D shows the ternary phase diagrams of the differences in rhizosphere soil bacteria and fungi, respectively, among the wheat, alfalfa, and maize/soybean rotation groups. After the difference analysis of all OTUs, five significantly different phyla were identified in both bacteria and fungi. However, these results could not fully classify the plant rhizosphere soil microorganisms according to the type of ground plant. This phenomenon suggests that although there are differences in these plant rhizosphere soil microorganisms, they had a high degree of homology, and conventional analysis methods could not fully reveal these differences.

#### 3.2.2. Soil Chemical Changes and Species Diversity Analysis

**Figure 3:**
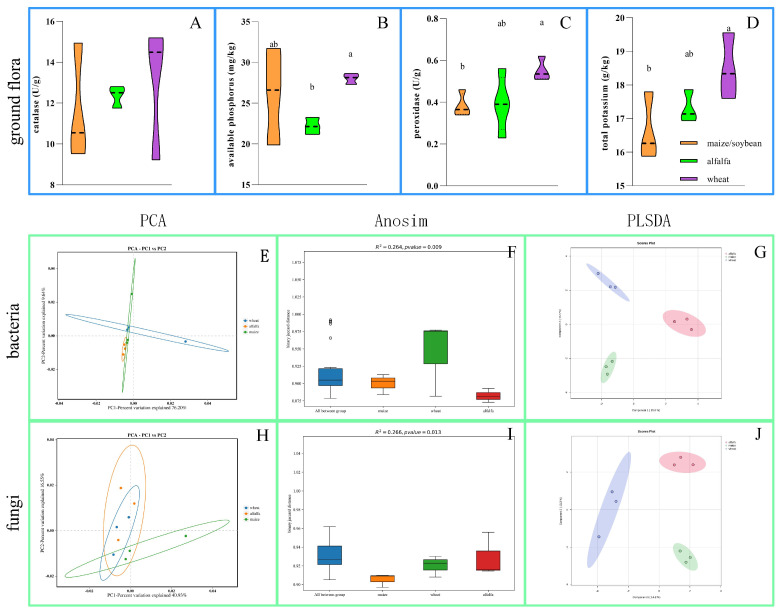
Soil chemical properties and plant rhizosphere soil microbial diversity analysis. (**A**–**D**) Catalase, available phosphorus, peroxidase, and total potassium content in soil planted with wheat (orange), alfalfa (green), and maize (purple). In the violin plots, different letters indicate significant differences. The PCA results for microbial diversity analysis for bacteria (**E**) and fungi (**H**) show overlapping areas, indicating an inability to distinguish between the groups. The Anosim results for bacteria ((**F**) *R*^2^ = 0.264; *p* = 0.009) and fungi ((**I**) *R*^2^ = 0.266; *p* = 0.013) reveal significant differences among the groups. The PLSDA results for bacteria (**G**) and fungi (**J**) clearly differentiate between wheat (poaceae), alfalfa (legume), and the maize and soybean rotation (control).

As shown in Figure 3A–D, after five years of cultivation of leguminous (alfalfa) and poaceae (wheat) plants, we observed changes in soil chemical compositions. Specifically, the contents of catalase (Figure 3C) and total potassium (Figure 3D) in the wheat soil were significantly higher than those in the maize and soybean rotation soil (control). At the same time, the available phosphorus (Figure 3C) and total potassium (Figure 3D) in the alfalfa (legume) soil were significantly higher than those in the maize and soybean rotation soil (control). The available phosphorus in the alfalfa soil was significantly lower than that in the wheat soil (Figure 3B). Soil microbial species diversity analysis showed that PCA failed to clearly distinguish the groups of wheat, alfalfa, and maize, as shown by the overlapping areas in Figure 3E,H. Anosim revealed significant differences in soil species diversity among the three crops (bacteria *p* = 0.009 and fungi *p* = 0.013) (Figure 3F,I). PLSDA also confirmed this, with the analysis results for bacteria (Figure 3G) and fungi (Figure 3J) clearly distinguishing wheat, alfalfa, and maize. These results suggest that although there are differences in the species diversity of soil in wheat, alfalfa, and maize, these differences are very limited.

#### 3.2.3. Plant and Microbial Coevolution

**Figure 4:**
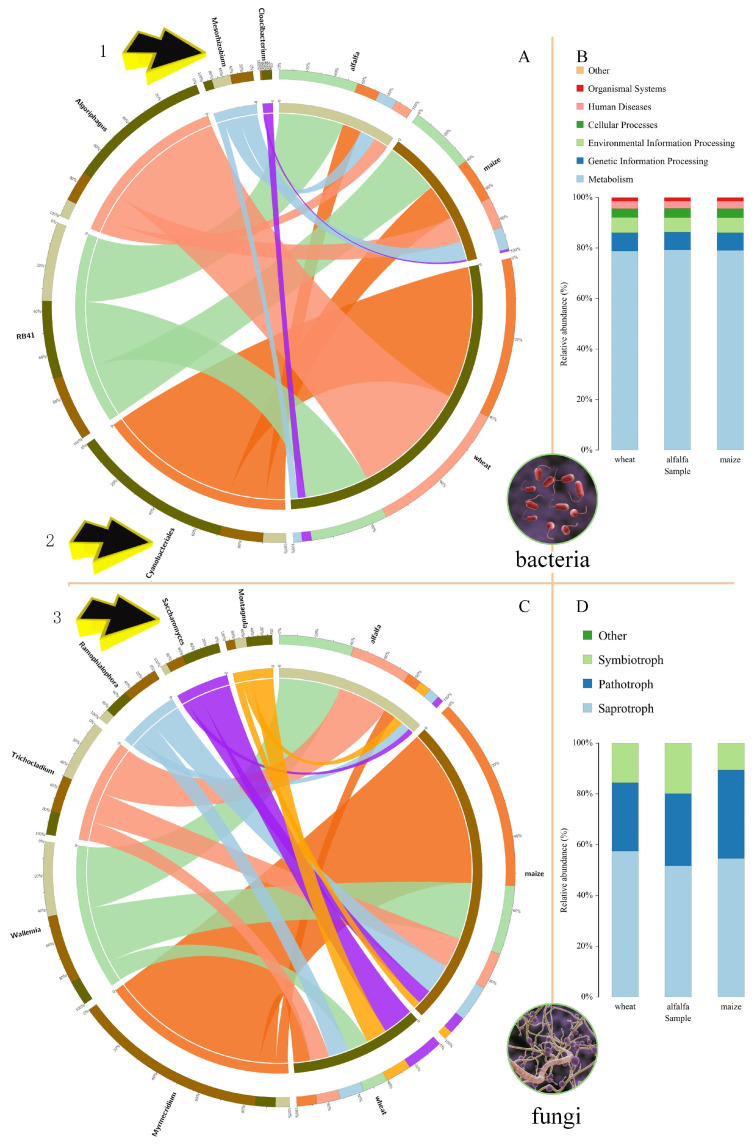
Circular charts of species composition and functional gene prediction. (**A**) Circular chart of the composition of soil rhizosphere bacteria. The left side of this chart represents the rhizosphere soil bacteria, while the right side represents wheat (poaceae), alfalfa (legume), and the maize and soybean rotation (control). The colored bands represent bacteria, with the width of the color indicating the relative abundance in the sample—the thicker the band, the richer the content of the species. Arrow 1 represents *Mesorhizobium*, and Arrow 2 represents *Cyanobacteriales*. (**B**) Functional gene prediction for soil rhizosphere bacteria. Different colors in the chart represent various KEGG annotation results. (**C**) Circular chart of soil rhizosphere fungi composition. Arrow 3 represents *Saccharomyces*. (**D**) Functional gene prediction for soil rhizosphere fungi.

Rhizosphere microorganisms change with the type of surface plants. Figure 4A shows the distribution of some of the previously mentioned soil rhizosphere bacteria in wheat, alfalfa, and maize. The relative abundance of *Rhizobia* (*Mesorhizobium*) was higher in the leguminous plants (alfalfa and soybean–maize rotation) than in the poaceae plants (wheat) (Arrow 1). The relative abundance of Cyanobacteriales was higher in the poaceae plants than in the leguminous plants (Arrow 2). The relative abundance of *RB41* in the roots of the three crops was very similar. Figure 4B shows the results of the functional gene prediction of soil rhizosphere bacteria. About 80% of the bacterial genes in the roots of the three crops were related to metabolism. Figure 4C describes the distribution of soil rhizosphere fungi in wheat, alfalfa, and maize. The relative abundance of *Saccharomyces* was higher in the wheat than in the leguminous plants (alfalfa and soybean–maize rotation) (Arrow 2). Figure 4D shows the results of the functional gene prediction of soil rhizosphere bacteria. About 80% of fungal genes were related to saprotrophs, suggesting that the nutritional source of fungi may depend mainly on the decay of straw and plant roots.

### 3.3. Correlation Analysis of Environmental Factors and Microorganisms

**Figure 5:**
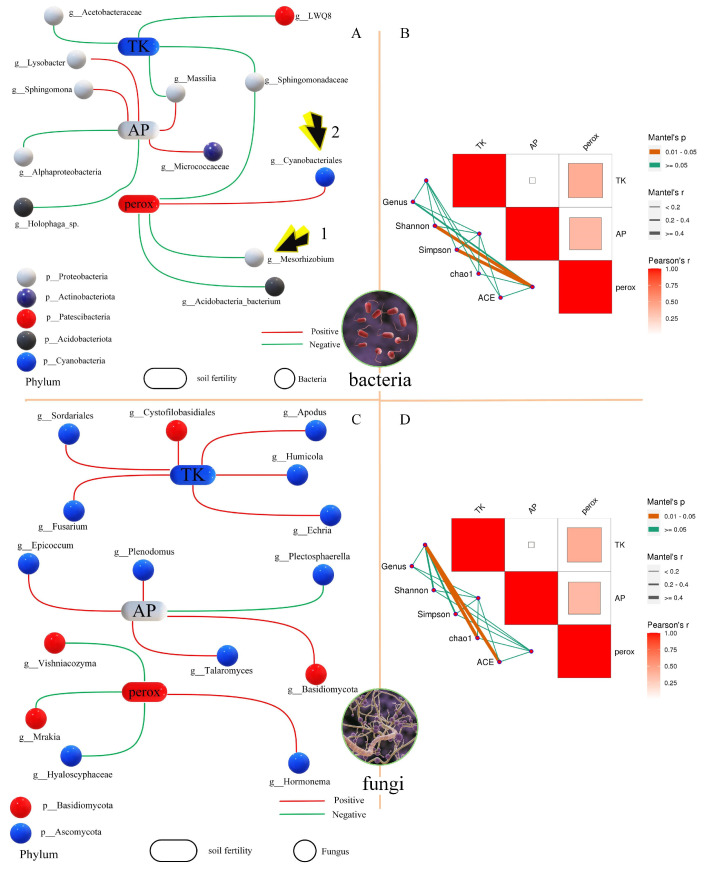
Heatmap and network combinatorial diagram of the correlation between environmental factors and surface plants and alpha index. (**A**) Correlation network diagram between soil fertility and bacteria. Arrow 1 represents *Mesorhizobium*, and Arrow 2 represents *Cyanobacteriales*. Balls of the same color represent the same phylum. Red lines indicate positive correlations, and green lines indicate negative correlations. (**B**) The heatmap and network combinatorial diagram for bacteria. Mantel’s *p* is the *p*-value of the correlation between environmental factors and bacteria and the Alpha index analyzed using a Mantel test; Mantel’s *r* is the r-value of the correlation between environmental factors and bacteria and the Alpha index analyzed using a Mantel test; and Pearson’s *r* is the r-value of the correlation between environmental factors and bacteria and the Alpha index. The heatmap in the upper right corner shows the correlation between environmental factors. The colors red and blue in the heatmap represent positive and negative correlations, respectively. The size of the heatmap block is consistent with the size of the correlation r. The network diagram in the lower left corner shows the network relationship between bacteria, the Alpha index, and environmental factors. The color of the lines is consistent with Mantel’s p in the legend, and the thickness of the lines is consistent with Mantel’s r in the legend. (**C**) The network combinatorial diagram of the correlation between soil fertility and fungi. Perox represents peroxidase, TK represents total potassium, and AP represents available phosphorus. g_ represents Genus. (**D**) The heatmap and network combinatorial diagram for fungi.

In this study, we comprehensively analyzed soil samples under the planting conditions of wheat (poaceae), alfalfa (legume), and a maize and soybean rotation (control) and considered peroxidase, total potassium, and available phosphorus as environmental factors. Figure 5A shows the correlation network diagram between soil fertility and bacteria. *Rhizobia* (*Mesorhizobium*) was negatively correlated with peroxidase, while Cyanobacteriales were positively correlated with peroxidase. In addition, peroxidase was positively correlated with the Shannon and Simpson indices of bacteria (Figure 5B). Figure 5C reveals the correlation network diagram between soil fertility and bacteria, with *Fusarium* being positively correlated with the total potassium content in the soil. Total potassium was also positively correlated with the chao1 and ACE richness indices of the fungal community (Figure 5D). There were also correlations between soil fertility indicators. Both the total potassium and available phosphorus were positively correlated with peroxidase (Figure 5B,D).

### 3.4. Peroxidase Protein 3D Structure Docking with L-Glutathione

**Figure 6:**
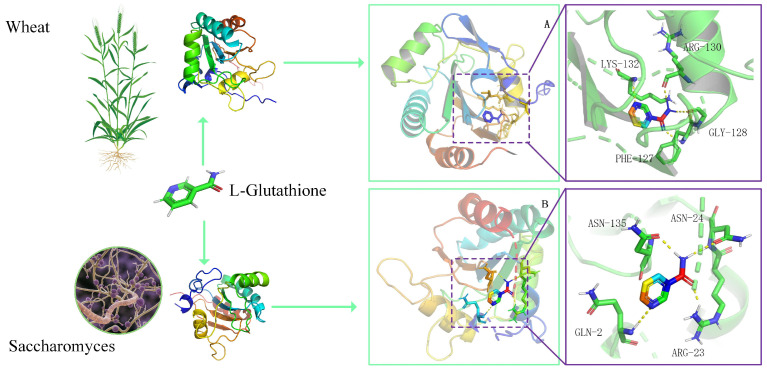
Peroxidase protein 3D structure docking with L-glutathione. (**A**) The 3D structure of the wheat peroxidase protein, consisting of 187 amino acids. The blue dashed box indicates the docking pocket. (**B**) The 3D structure of the *Saccharomyces* peroxidase protein, consisting of 167 amino acids, with 504 encoded bases. The yellow dashed line represents a connection bond of less than 5 Å. This image was rotated to keep the docking pocket clearly visible, with the amino acids not directly connected to L-glutathione hidden.

In this study, we compared and analyzed the molecular characteristics of wheat and *Saccharomyces* glutathione peroxidase, the number of amino acids, the three-dimensional structure, and the differences in their active site pockets. Wheat glutathione peroxidase had a docking pocket composed of LYS-132, ARG-130, PHE-127, and GLY-128, with a global match quality estimate value (GMQE) of 0.959 (Figure 6A). In contrast, the docking pocket of *Saccharomyces* glutathione peroxidase was composed of ASN-135, ASN-24, GLN-2, and ARG-23, with a GMQE value of 0.901 (Figure 6B). Despite the different structures of the docking pockets, the binding affinity of glutathione peroxidase from wheat and brewer’s yeast to the substrate L-glutathione showed similarity, at −5.1 and −5.3 kcal/mol, respectively.

## 4. Discussion

This study found that soil microbial communities were affected by human cultivation activities. This research discovered a coevolutionary relationship between the structure of plant rhizosphere soil microbial communities and the plants planted on the surface. With the continuous growth of the global population, humans have been seeking various ways to increase grain yields to meet the increasing burden of the world population [1]. Changing the soil microbial community has become a hot field of research. The use of beneficial Arbuscular Mycorrhizal Fungi (AMF) to plants has been proven to increase crop yields [26,27] and enhance plant resistance to heavy metal stress [28,29], salt stress [26,30,31], and plant diseases [32,33]. As a typical plant symbiotic fungus, AMF enter the plant’s roots through its hyphae, exchange nutrients with the plant, and expand the plant’s rhizosphere [27,34]. In particular, there is also a coevolutionary relationship between AMF and *Rhizobia* [35].

### 4.1. Correlation of Rhizobia and Peroxidase in Soil

The common view is that soil peroxidase is closely related to disease and stress resistance in plants [14,15]. However, the results of this study show that endogenous *Rhizobia* (*Mesorhizobium*) in the soil was negatively correlated with peroxidase (Figure 5A). Benidire (2021) also reported similar results, pointing out that if 100% agricultural soil was used and *Rhizobia* were added, the peroxidase content in the soil was significantly reduced [14]. This result is consistent with our findings. However, many published studies have shown that the addition of exogenous *Rhizobia* can increase the peroxidase content in the soil [11,14]. To explore the reasons for this phenomenon, we conducted a comparative analysis of the published literature.

A recent study addressing soil amelioration of mine tailings offered new insights. Using 100% agricultural soil and adding *Rhizobia* resulted in a significant decrease in peroxidase levels (*p* < 0.05). In contrast, a mixture of agricultural soil, mine tailings, compost, and either rock phosphate or CaCO_3_ led to a significant increase in peroxidase levels (*p* < 0.05) [14]. Furthermore, in the case of chickpea (*Cicer arietinum* L.) challenged by *Fusarium* wilt, the addition of exogenous *Rhizobia*-based biofilms resulted in an increase in soil peroxidase levels over time. This process remained constant for the first 7 days, began to rise on the 14th day, peaked on the 21st day with the highest peroxidase levels, and then declined by the 28th day [11].

In addition to the specific beneficial *Rhizobia* discussed above, a diverse range of *Rhizobia* exists in nature, with those in the plant rhizosphere classified into 15 genera [10]. Moreover, fertilization practices can also lead to changes in *Rhizobia* abundance, with the long-term application of organic fertilizers increasing the *Rhizobia* content in the soil [8]. The application of nitrogen and phosphorus-deficient fertilizers can reduce or result in ineffective root nodules in the roots of chickpea (*Cicer arietinum* L.). The results of this study also indicate that the available phosphorus content in the soil of alfalfa (legume) was significantly lower than that in wheat (poaceae) (Figure 3B). Therefore, this study employed field trial methods (with 100% agricultural soil; Figure 1A), where the nitrogen fertilizer used for the leguminous plants was significantly lower than that for the poaceae plants (Table 1), and soil samples were collected at the end of the plant growth cycle in the fall. The combination of these factors led to the observed negative correlation between *Rhizobia* (*Mesorhizobium*) and oxidase enzyme activity (Figure 5A).

### 4.2. Characteristics of Rhizosphere Microorganisms of Leguminous and Poaceae Plants

The root cortex of leguminous plants contains a key signaling initiation mechanism controlled by the *SHORTROOT-SCARECROW* (*SHR-SCR*) gene module, which can respond to *Rhizobia*, thereby inducing root nodule formation [36]. Subsequently, in soybeans, the *GmNLP* (*NIN-LIKE PROTEIN*) and *GmTCP* (*TEOSINTE-BRANCHED1/CYCLOIDEA/PCF*) genes regulate the nodule phenotype according to the soil nitrogen concentration [37]. During this process, *Rhizobia* secretes a protein, NopT, that binds to the soybean kinase GmPBS1, thereby regulating the development of symbiotic nodules through the AtPBS1/AtRPS5 resistance pathway [38].

**Figure 7:**
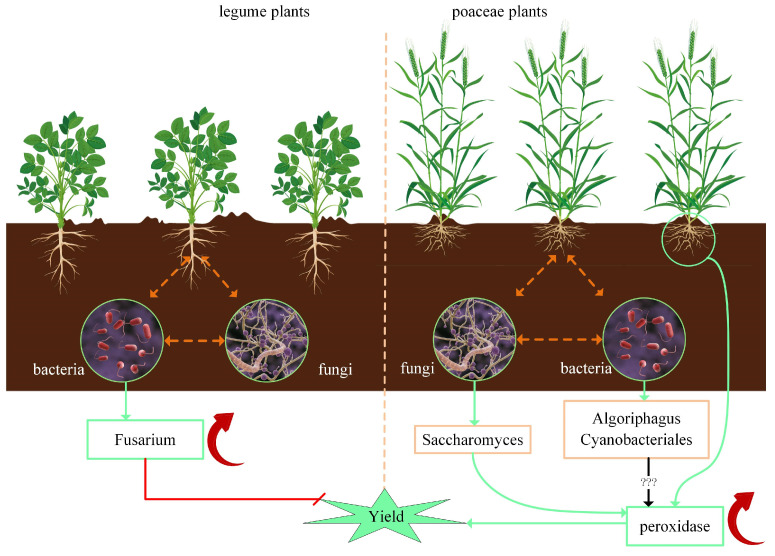
Interactions between rhizosphere microorganisms and plants. Surface plants, plant rhizosphere, and bacteria and fungi in the soil form a close symbiotic environment, coevolving within this system. Continuous cultivation of leguminous plants increases the abundance of *Fusarium* in the soil, thereby affecting yields. Continuous cultivation of wheat (poaceae) can increase the abundance of *Saccharomyces* in the rhizosphere, thereby increasing the content of soil peroxidase and ultimately increasing the wheat yield. Continuous wheat cultivation can also increase the abundance of Cyanobacteriales and *Algoriphagus* in the rhizosphere. However, no studies have reported a correlation between Cyanobacteriales and the soil peroxidase content.

In agricultural ecosystems, soil microorganisms can be categorized based on their functions into harmful and beneficial microorganisms [13]. The enrichment of pathogenic fungi in crops can lead to crop diseases. Continuous cropping of leguminous crops can result in reduced yields (Figure 7). The continuous cultivation of soybeans leads to changes in the composition of the rhizosphere microbial community, which, in turn, reduces yields [39,40,41]. Studies have confirmed that continuous cropping of soybeans alters the types of fungi, significantly increasing the amount of *Thanatophorus* and *Fusarium*, causing diseases to occur [42,43]. In this study, although *Fusarium* was found to be positively correlated with the total potassium content in the soil (Figure 5C), no occurrence of *Fusarium* disease was observed throughout the experimental process. Generally, once alfalfa is planted, it can be used for five years. The results of this experiment also show that alfalfa reached its yield peak in the third year of cultivation (Figure 1C), and then, the yield significantly decreased in the following two years (2022 and 2023). We speculate that this may be related to the cultivation of soybeans in the year before alfalfa planting (2018).

It is widely accepted that soil peroxidase is closely related to the disease resistance and stress tolerance of plants [14,15]. This study indicates that the relative abundance of *Saccharomyces* in the rhizosphere soil of wheat (poaceae) is significantly higher than that of the maize and soybean rotation (control) and alfalfa (legume) (Figure 4C). Studies have shown that *Saccharomyces* is positively correlated with peroxidase content [16,17]. In crude-oil polluted soil, *Saccharomyces* can increase the peroxidase content, playing an active role in soil remediation [16]. The addition of *Saccharomyces* during the cultivation process of faba beans not only effectively inhibits root rot diseases but also increases the soil peroxidase content [17]. The results of this study are similar, showing that after the coevolution of wheat and soil microorganisms, the yield increased significantly (Figure 1C). In addition, this study found that in the rhizosphere soil of wheat, the relative abundance of Cyanobacteriales and *Algoriphagus* was significantly higher than that of maize and alfalfa (Figure 4A and Figure 7), but the reasons for this phenomenon have not been fully explained in the published literature to date. Advanced simulations of bacterial protein structures can reveal the metabolic characteristics of genes [44]. This study simulated the advanced structures of peroxidase proteins in wheat and *Saccharomyces* and compared the docking results with L-glutathione (Figure 6), suggesting that the two are very similar in terms of function and catalytic ability. Whether wheat and *Saccharomyces* can jointly utilize soil peroxidase requires further research.

### 4.3. Plants and Rhizosphere Microbial Fertilizers

Given the important role of rhizosphere microorganisms in promoting plant growth [45], enhancing plant disease resistance [32,33], and strengthening plant stress tolerance [28,29], there are already commercial rhizosphere probiotic fertilizers applied in agricultural production [46]. Among these rhizosphere microbial probiotic fertilizers, the more common types include AMF [26,27], *Bacillus subtilis* [47], *Bacillus mucilaginosus* [48], *Bacillus licheniformis* [49,50,51], *Bacillus amyloliquefaciens* [52], *Saccharomyces* [14,15,53], and *Lactobacillus plantarum* [54].

The latest research indicates that about 90% of rhizosphere microorganisms reside in biofilms [55]. *Bacillus subtilis* has been found to aggregate at the roots of plants to form a biofilm, and the formation of this biological film can further exert biological functions [56]. This process of colonization involves complex and precise two-way chemical communication between multiple microorganisms and plant roots [57]. Furthermore, *Bacillus subtilis* can secrete Spermidine, which, after entering the plant root system, increases the expression level of plant glutathione enzymes, thereby reducing the damage caused by reactive oxygen species (ROS) under salt stress conditions, ultimately promoting plant growth [58]. In addition, *Bacillus subtilis* secretes a variety of lipopeptides and polyketide antibiotics, which help protect plants from pathogen attacks [59,60].

### 4.4. Coevolution of Plants and Rhizosphere Microorganisms

Based on the results of this study, combined with other published papers, we summarized the interaction between rhizosphere microorganisms and plants (Figure 7). The most frequently reported interaction between plants and microorganisms is the symbiotic relationship between soybeans and *Rhizobia* [9,10,61]. This is a typical symbiotic nitrogen-fixing action in which many *Rhizobia* species can convert atmospheric nitrogen (N_2_) into ammonium (NH^4+^) that plants can utilize [62,63]. Soybeans, in turn, provide the organic matter needed by *Rhizobia*, such as carbohydrates [12]. This mutually beneficial relationship allows soybeans to grow well in soils that are relatively nitrogen-deficient. However, the unreasonably excessive application of nitrogen fertilizers can lead to nitrogen volatilization into the atmosphere [63]. In this study, the amount of nitrogen fertilizer used for the leguminous plants was lower than that for the poaceae plants (Table 1). Despite this, continuous cropping of leguminous plants increases the amount of *Fusarium*, leading to pathogen emergence [42,43], which results in reduced yields (Figure 7).

In nature, the coevolution between plants and rhizosphere microbes can enhance drought resistance, giving the Rhizobiont a higher chance of survival in water-deficient environments [64]. Similar results indicate that this coevolution helps maintain plant health [65], protects the host from diseases [66], and sustains a healthy soil cycle [67,68]. Therefore, studying the Rhizobiont is of key significance in supporting agricultural sustainability [69], especially in the process of plants facing various biotic and abiotic stressors and adapting to environmental changes [70].

The aforementioned studies demonstrate synergistic actions within the Rhizobiont. However, the most critical aspect is that the plant’s root is the foundation that determines everything. Human agricultural practices determine the types of crops planted, thereby fundamentally and continuously altering the Rhizobiont. This was also the original intention of this study.

### 4.5. Human Activities Determine the Rhizobiont

Ultimately, this study posits that under human cultivation conditions, the coevolution of rhizosphere microbes is a passive response mechanism to meet the survival requirements of the microbes. Humans, by deciding the types of plants grown on the surface, fundamentally change biodiversity under natural conditions. To increase crop yields, people often rely on the use of chemical fertilizers and refined field management, without considering the destruction of rhizosphere microbial communities. Based on the response of rhizosphere microbes to agricultural cultivation conditions, this study categorizes soil microbes under agricultural cultivation into three types: adaptive microbes, neutral microbes, and resistant microbes. This is a broad and somewhat vague definition, because it needs to be combined with specific types of surface plants for a detailed explanation.

Adaptive microbes can effectively utilize chemical fertilizers or form symbiotic relationships with surface plants, with the notable characteristic of increasing their population size. It is also possible that human cultivation has made the soil environment more suitable for the growth of adaptive microbes. For example, rice cultivation leads to a relatively higher abundance of anaerobic bacteria in soil [71]. According to the results of this study, *Saccharomyces* is a typical adaptive microbe when wheat is cultivated.

Neutral microbes cannot effectively utilize fertilizers or land management methods; their population size gradually decreases or even becomes extinct in competition with other microbes. The vast majority of soil microbes belong to this type, including those we have not yet discovered that interact with plants. For instance, *RB41* is currently a neutral microbe in wheat, alfalfa, and maize, and its population size has not significantly changed due to agricultural activities.

Resistant microbes can also utilize fertilizers and land management methods provided by agricultural cultivation, but they directly or indirectly inhibit the growth of surface plants. In this study, *Fusarium* is a typical resistant microbe when soybeans are cultivated. A notable characteristic of resistant microbes is the suppression of surface plant growth. However, this is not based on changes in their relative abundance but serves as a regulatory mechanism in nature to maintain species diversity. Rhizosphere microbes play an important role in ecosystems by driving plant populations and community ecological processes [72].

In the discussion of soil microbial classification, a microbe can have multiple labels. For example, *Fusarium* is a typical resistant microbe when cultivating soybeans, but it also shows the characteristics of adaptive microbes (Figure 7). However, the labels of adaptive microbes and neutral microbes are mutually exclusive; in other words, a microbe cannot be classified as either adaptive or neutral at the same time.

#### Limitations

This study compared the binding capacity of glutathione peroxidase proteins from wheat and *Saccharomyces* with the ligand L-glutathione. Our results suggest that these two peroxidases may have potentially similar roles in ligand binding. However, the mere binding capacity is not sufficient to fully represent the catalytic activity of the enzyme. This limitation means that although the two proteins show certain similarities in their binding characteristics to the ligand, this does not guarantee that they have the same or similar efficiency and kinetic properties in the catalytic reaction.

## 5. Conclusions

Human agricultural cultivation activities have changed the composition of surface plants, which inevitably affects the Rhizobiont in the soil. This study aimed to explore the survival and reproduction strategies of soil microbes under the pressure of human agricultural cultivation: whether to resist or adapt. Based on this, we categorized soil microbes under agricultural cultivation conditions into three types: adaptive microbes, neutral microbes, and resistant microbes. Adaptive microbes can effectively utilize chemical fertilizers or establish symbiotic relationships with plants. In contrast, resistant microbes, although they can also utilize fertilizers and land management methods of human agricultural cultivation, directly or indirectly inhibit the growth of surface plants. For instance, when wheat is continuously cultivated, *Saccharomyces* is a typical adaptive microbe; when soybeans are continuously cultivated, *Fusarium* is a typical resistant microbe. However, focusing on the microbial ecology of the plant rhizosphere, the plant rhizosphere soil microbial community maintains a coevolutionary relationship with human cultivation activities. Future research is needed to further understand the adaptability of soil microbial communities to agricultural cultivation to confirm the impact of the massive growth of agricultural cultivation-adapted communities in soil microbes on the global ecosystem.

## Figures and Tables

**Table 1:** Fertilizer application rates in different experimental groups.

Crop	Urea (kg/h × m^2^)	Diammonium Phosphate (kg/h × m^2^)	Potassium Sulfate (kg/h × m^2^)
Soybean	25	150	65
Maize	250	150	75
Wheat	100	163	75
Alfalfa	50	200	150

## Data Availability

The original contributions presented in this study are included in the article. Further inquiries can be directed to the corresponding authors.

## Abbreviations

The following abbreviations are used in this manuscript:

ANOVA	Analysis of variance
OTUs	Operational taxonomic units
PLSDA	Partial Least Squares Discrimination Analysis
GMQE	Global match quality estimate value
AMF	Arbuscular Mycorrhizal Fungi

## References

[1] Riris P., Silva F., Crema E., Palmisano A., Robinson E., Siegel P.E., French J.C., Jorgensen E.K., Maezumi S.Y., Solheim S. (2024). Frequent disturbances enhanced the resilience of past human populations. Nature.

[2] Peters M.K., Hemp A., Appelhans T., Becker J.N., Behler C., Classen A., Detsch F., Ensslin A., Ferger S.W., Frederiksen S.B. (2019). Climate-land-use interactions shape tropical mountain biodiversity and ecosystem functions. Nature.

[3] Hong C., Burney J.A., Pongratz J., Nabel J., Mueller N.D., Jackson R.B., Davis S.J. (2021). Global and regional drivers of land-use emissions in 1961–2017. Nature.

[4] Springmann M., Clark M., Mason-D’Croz D., Wiebe K., Bodirsky B.L., Lassaletta L., de Vries W., Vermeulen S.J., Herrero M., Carlson K.M. (2018). Options for keeping the food system within environmental limits. Nature.

[5] Tian J., Wang C., Chen F., Qin W., Yang H., Zhao S., Xia J., Du X., Zhu Y., Wu L. (2024). Maize smart-canopy architecture enhances yield at high densities. Nature.

[6] Zhu Y., Qu Z., Zhao J., Wang J., Wei D., Meng Q. (2024). Can high-yielding maize system decrease greenhouse gas emissions largely while simultaneously enhancing economic and ecosystem benefits through the “Rhizobiont” concept? Evidence from field. Sci. Total Environ..

[7] Stone B.W., Li J., Koch B.J., Blazewicz S.J., Dijkstra P., Hayer M., Hofmockel K.S., Liu X.A., Mau R.L., Morrissey E.M. (2021). Nutrients cause consolidation of soil carbon flux to small proportion of bacterial community. Nat. Commun..

[8] Bian Q., Cheng K., Chen L., Jiang Y., Li D., Xie Z., Wang X., Sun B. (2024). Organic amendments increased Chinese milk vetch symbiotic nitrogen fixation by enriching Mesorhizobium in rhizosphere. Environ. Res..

[9] Nahusenay G., Wolde G., Tena W., Tamiru T. (2024). Chickpea (*Cicer arietinum* L.) growth, nodulation, and yield as affected by varieties, Mesorhizobium strains, and NPSB fertilizer in Southern Ethiopia. Front. Plant Sci..

[10] Li Y., Guo T., Sun L., Wang E.T., Young J.P.W., Tian C.F. (2024). Phylogenomic analyses and reclassification of the Mesorhizobium complex: Proposal for 9 novel genera and reclassification of 15 species. BMC Genom..

[11] Das K., Rajawat M.V.S., Saxena A.K., Prasanna R. (2017). Development of Mesorhizobium ciceri-Based Biofilms and Analyses of Their Antifungal and Plant Growth Promoting Activity in Chickpea Challenged by Fusarium Wilt. Indian J. Microbiol..

[12] Mayhood P., Mirza B.S. (2021). Soybean Root Nodule and Rhizosphere Microbiome: Distribution of Rhizobial and Nonrhizobial Endophytes. Appl. Environ. Microbiol..

[13] Liu H., Pan F., Han X., Song F., Zhang Z., Yan J., Xu Y. (2018). Response of Soil Fungal Community Structure to Long-Term Continuous Soybean Cropping. Front. Microbiol..

[14] Benidire L., Madline A., Pereira S.I.A., Castro P.M.L., Boularbah A. (2021). Synergistic effect of organo-mineral amendments and plant growth-promoting rhizobacteria (PGPR) on the establishment of vegetation cover and amelioration of mine tailings. Chemosphere.

[15] Hohenfeld C.S., de Oliveira S.A.S., Ferreira C.F., Mello V.H., Margarido G.R.A., Passos A.R., de Oliveira E.J. (2024). Comparative analysis of infected cassava root transcriptomics reveals candidate genes for root rot disease resistance. Sci. Rep..

[16] Okeke P., Onyeocha I., Ezeji U., Chukwudi P., Engwa G. (2020). Biodegradation of hydrocarbons in a crude-oil polluted soil using peroxidase from fungal di-culture of *Rhizopus* and *Saccharomyces* spp. J. Biotech. Res..

[17] El-Sayed S. (2022). Collaborative Potentialities of *Trichoderma* spp. and *Saccharomyces cerevisiae* Against Damping-off and Root Rot Diseases of Faba Bean. Egypt. J. Phytopathol..

[18] Aiyer H.S., McKenzie-Gopsill A., Mills A., Foster A.J. (2024). Select Cover Crop Residue and Soil Microbiomes Contribute to Suppression of Fusarium Root and Crown Rot in Barley and Soybean. Microorganisms.

[19] Rafi N., Dominguez M., Okello P.N., Mathew F.M. (2024). No Common Candidate Genes for Resistance to *Fusarium graminearum*, *F. proliferatum*, *F. sporotrichioides*, and *F. subglutanins* in Soybean (*Glycine max* L.) Accessions from Maturity Groups 0 and I: Findings from Genome-Wide Association Mapping. Plant Dis..

[20] Shi X., Yang G., Yu D., Xu S., Warner E.D., Petersen G.W., Sun W., Zhao Y., Easterling W.E., Wang H. (2010). A WebGIS system for relating genetic soil classification of China to soil taxonomy. Comput. Geosci..

[21] Wang Q., Liu X., Li J., Li P., Zuo X., Chang B., Liu Y., Zhang N., Yu H., Miao L. (2023). Temporal variation in soil carbon in various paddy soil types in a cold temperate continental monsoon climate. Soil. Use Manag..

[22] (2006). Soil Testing. Part 1: Soil Sampling, Processing and Reposition.

[23] Lin D. (2004). Guidance of Soil Science Experiment.

[24] (2012). Soil Testing. Part 24: Determination of Total Nitrogen in Soil. Automatic Kjeldahl Apparatus Method.

[25] Chen Y., Shan X., Jiang H., Sun L., Guo Z. (2023). Regulation of litter size in sheep (*Ovis aries*) by the GDF9 and BMP15 genes. Ann. Agric. Sci..

[26] de Carvalho Neta S.J., Araujo V., Fracetto F.J.C., da Silva C.C.G., de Souza E.R., Silva W.R., Lumini E., Fracetto G.G.M. (2024). Growth-promoting bacteria and arbuscular mycorrhizal fungus enhance maize tolerance to saline stress. Microbiol. Res..

[27] Li M.Y., Wang W., Mo F., Ren A.T., Wang Z.Y., Zhu Y., Xiong Y.C. (2024). Seven-year long-term inoculation with *Funneliformis mosseae* increases maize yield and soil carbon storage evidenced by in situ (13)C-labeling in a dryland. Sci. Total Environ..

[28] Becerra A.G., Menoyo E., Faggioli V., Cabello M., Salazar M.J. (2023). Mycorrhizal fungal communities associated with three metal accumulator plants growing in an abandoned Pb smelting factory. Braz. J. Microbiol..

[29] Jia Q., Sun J., Gan Q., Shi N.N., Fu S. (2024). Zea mays cultivation, biochar, and arbuscular mycorrhizal fungal inoculation influenced lead immobilization. Microbiol. Spectr..

[30] Barajas Gonzalez J.A., Carrillo-Gonzalez R., Gonzalez-Chavez M., Chimal Sanchez E., Tapia Maruri D. (2023). Selection of Salinity-Adapted Endorhizal Fungal Consortia from Two Inoculum Sources and Six Halophyte Plants. J. Fungi.

[31] Huang P., Huang S., Ma Y., Danish S., Hareem M., Syed A., Elgorban A.M., Eswaramoorthy R., Wong L.S. (2024). Alleviation of salinity stress by EDTA chelated-biochar and arbuscular mycorrhizal fungi on maize via modulation of antioxidants activity and biochemical attributes. BMC Plant Biol..

[32] Yagmur A., Demir S., Canpolat S., Rezaee Danesh Y., Farda B., Djebaili R., Pace L., Pellegrini M. (2024). Onion *Fusarium* Basal Rot Disease Control by Arbuscular Mycorrhizal Fungi and *Trichoderma harzianum*. Plants.

[33] Yang Z., Kang J., Ye Z., Qiu W., Liu J., Cao X., Ge J., Ping W. (2023). Synergistic benefits of *Funneliformis mosseae* and *Bacillus paramycoides*: Enhancing soil health and soybean tolerance to root rot disease. Environ. Res..

[34] Khan W., Zhu Y., Khan A., Zhao L., Yang Y.M., Wang N., Hao M., Ma Y., Nepal J., Ullah F. (2024). Above-and below-ground feedback loop of maize is jointly enhanced by plant growth-promoting rhizobacteria and arbuscular mycorrhizal fungi in drier soil. Sci. Total Environ..

[35] Wang X., Feng H., Wang Y., Wang M., Xie X., Chang H., Wang L., Qu J., Sun K., He W. (2021). Mycorrhizal symbiosis modulates the rhizosphere microbiota to promote rhizobia-legume symbiosis. Mol. Plant.

[36] Dong W., Zhu Y., Chang H., Wang C., Yang J., Shi J., Gao J., Yang W., Lan L., Wang Y. (2021). An SHR-SCR module specifies legume cortical cell fate to enable nodulation. Nature.

[37] Kim Y., Wang J., Ma C., Jong C., Jin M., Cha J., Wang J., Peng Y., Ni H., Li H. (2023). GmTCP and GmNLP Underlying Nodulation Character in Soybean Depending on Nitrogen. Int. J. Mol. Sci..

[38] Khan A., Wadood S.F., Chen M., Wang Y., Xie Z.P., Staehelin C. (2022). Effector-triggered inhibition of nodulation: A rhizobial effector protease targets soybean kinase GmPBS1-1. Plant Physiol..

[39] He D., Yao X., Zhang P., Liu W., Huang J., Sun H., Wang N., Zhang X., Wang H., Zhang H. (2023). Effects of continuous cropping on fungal community diversity and soil metabolites in soybean roots. Microbiol. Spectr..

[40] Mengistu A., Read Q.D., Sykes V., Kelly H., Kharel T., Bellaloui N. (2024). Cover Crop and Crop Rotation Effects on Tissue and Soil Population Dynamics of *Macrophomina phaseolina* and Yield Under No-Till System. Plant Dis..

[41] Wu D., Zhang Y., Gu W., Feng Z., Xiu L., Zhang W., Chen W. (2024). Long term co-application of biochar and fertilizer could increase soybean yield under continuous cropping: Insights from photosynthetic physiology. J. Sci. Food Agric..

[42] Perez-Brandan C., Huidobro J., Grumberg B., Scandiani M.M., Luque A.G., Meriles J.M., Vargas-Gil S. (2014). Soybean fungal soil-borne diseases: A parameter for measuring the effect of agricultural intensification on soil health. Can. J. Microbiol..

[43] Kang I.J., Lee M., Han S.Y., Kim Y.H., Lee S. (2024). First report of soybean root rot caused by *Fusarium proliferatum* in the Republic of Korea. Plant Dis..

[44] Zhao W., Zhong B., Zheng L., Tan P., Wang Y., Leng H., de Souza N., Liu Z., Hong L., Xiao X. (2022). Proteome-wide 3D structure prediction provides insights into the ancestral metabolism of ancient archaea and bacteria. Nat. Commun..

[45] Dai C., Shu Z., Ma C., Yan P., Huang L., He R., Ma H. (2024). Isolation of a surfactin-producing strain of *Bacillus subtilis* and evaluation of the probiotic potential and antioxidant activity of surfactin from fermented soybean meal. J. Sci. Food Agric..

[46] Islam T., Fatema, Hoque M.N., Gupta D.R., Mahmud N.U., Sakif T.I., Sharpe A.G. (2023). Improvement of growth, yield and associated bacteriome of rice by the application of probiotic *Paraburkholderia* and *Delftia*. Front. Microbiol..

[47] Abuhena M., Al-Rashid J., Azim M.F., Khan M.N.M., Kabir M.G., Barman N.C., Rasul N.M., Akter S., Huq M.A. (2022). Optimization of industrial (3000 L) production of *Bacillus subtilis* CW-S and its novel application for minituber and industrial-grade potato cultivation. Sci. Rep..

[48] Anusauskas J., Steponavicius D., Romaneckas K., Lekaviciene K., Zaleckas E., Sendzikiene E. (2023). The Influence of Bacteria-Inoculated Mineral Fertilizer on the Productivity and Profitability of Spring Barley Cultivation. Plants.

[49] Shukla A., Gupta A., Srivastava S. (2023). Bacterial consortium (Priestia endophytica NDAS01F, Bacillus licheniformis NDSA24R, and Priestia flexa NDAS28R) and thiourea mediated amelioration of arsenic stress and growth improvement of *Oryza sativa* L. Plant Physiol. Biochem..

[50] Rios-Ruiz W.F., Tuanama-Reategui C., Huaman-Cordova G., Valdez-Nunez R.A. (2023). Co-Inoculation of Endophytes *Bacillus siamensis* TUR07-02b and *Priestia megaterium* SMBH14-02 Promotes Growth in Rice with Low Doses of Nitrogen Fertilizer. Plants.

[51] Romero-Munar A., Aroca R. (2023). A non-K(+)-solubilizing PGPB (*Bacillus megaterium*) increased K(+) deprivation tolerance in *Oryza sativa* seedlings by up-regulating root K(+) transporters. Plant Physiol. Biochem..

[52] Liang M., Feng A., Wang C., Zhu X., Su J., Xu Z., Yang J., Wang W., Chen K., Chen B. (2024). *Bacillus amyloliquefaciens* LM-1 Affects Multiple Cell Biological Processes in *Magnaporthe oryzae* to Suppress Rice Blast. Microorganisms.

[53] Zveushe O.K., de Dios V.R., Zhang H., Zeng F., Liu S., Shen S., Kang Q., Zhang Y., Huang M., Sarfaraz A. (2023). Effects of Co-Inoculating *Saccharomyces* spp. with *Bradyrhizobium japonicum* on Atmospheric Nitrogen Fixation in Soybeans (*Glycine max* (L.)). Plants.

[54] Yin H., Zhao M., Yang R., Sun J., Yu Z., Bai C., Xue Y. (2024). Effect of Regulation of Whole-Plant Corn Silage Inoculated with *Lactobacillus buchneri* or *Bacillus licheniformis* Regarding the Dynamics of Bacterial and Fungal Communities on Aerobic Stability. Plants.

[55] Flemming H.C., van Hullebusch E.D., Neu T.R., Nielsen P.H., Seviour T., Stoodley P., Wingender J., Wuertz S. (2023). The biofilm matrix: Multitasking in a shared space. Nat. Rev. Microbiol..

[56] Zhou X., Zhang N., Xia L., Li Q., Shao J., Shen Q., Zhang R. (2018). ResDE Two-Component Regulatory System Mediates Oxygen Limitation-Induced Biofilm Formation by *Bacillus amyloliquefaciens* SQR9. Appl. Environ. Microbiol..

[57] Xu Z., Liu Y., Zhang N., Xun W., Feng H., Miao Y., Shao J., Shen Q., Zhang R. (2023). Chemical communication in plant-microbe beneficial interactions: A toolbox for precise management of beneficial microbes. Curr. Opin. Microbiol..

[58] Chen L., Liu Y., Wu G., Zhang N., Shen Q., Zhang R. (2017). Beneficial Rhizobacterium *Bacillus amyloliquefaciens* SQR9 Induces Plant Salt Tolerance through Spermidine Production. Mol. Plant Microbe Interact..

[59] Wu G., Liu Y., Xu Y., Zhang G., Shen Q., Zhang R. (2018). Exploring Elicitors of the Beneficial Rhizobacterium Bacillus amyloliquefaciens SQR9 to Induce Plant Systemic Resistance and Their Interactions with Plant Signaling Pathways. Mol. Plant Microbe Interact..

[60] Zhang N., Yang D., Wang D., Miao Y., Shao J., Zhou X., Xu Z., Li Q., Feng H., Li S. (2015). Whole transcriptomic analysis of the plant-beneficial rhizobacterium *Bacillus amyloliquefaciens* SQR9 during enhanced biofilm formation regulated by maize root exudates. BMC Genom..

[61] Hata S., Tsuda R., Kojima S., Tanaka A., Kouchi H. (2023). Both incompatible and compatible rhizobia inhabit the intercellular spaces of leguminous root nodules. Plant Signal Behav..

[62] Kim M., Kim W., Park Y., Jung J., Park W. (2024). Lineage-specific evolution of *Aquibium*, a close relative of *Mesorhizobium*, during habitat adaptation. Appl. Environ. Microbiol..

[63] Fudjoe S.K., Li L., Anwar S., Shi S., Xie J., Wang L., Xie L., Yongjie Z. (2023). Nitrogen fertilization promoted microbial growth and N_2_O emissions by increasing the abundance of nirS and nosZ denitrifiers in semiarid maize field. Front. Microbiol..

[64] Hestrin R., Kan M., Lafler M., Wollard J., Kimbrel J.A., Ray P., Blazewicz S.J., Stuart R., Craven K., Firestone M. (2022). Plant-associated fungi support bacterial resilience following water limitation. ISME J..

[65] Getzke F., Thiergart T., Hacquard S. (2019). Contribution of bacterial-fungal balance to plant and animal health. Curr. Opin. Microbiol..

[66] Perez-de-Luque A., Tille S., Johnson I., Pascual-Pardo D., Ton J., Cameron D.D. (2017). The interactive effects of arbuscular mycorrhiza and plant growth-promoting rhizobacteria synergistically enhance host plant defences against pathogens. Sci. Rep..

[67] Saleem M., Hu J., Jousset A. (2019). More Than the Sum of Its Parts: Microbiome Biodiversity as a Driver of Plant Growth and Soil Health. Annu. Rev. Ecol. Evol. Syst..

[68] Cao T., Fang Y., Chen Y., Kong X., Yang J., Alharbi H., Kuzyakov Y., Tian X. (2022). Synergy of saprotrophs with mycorrhiza for litter decomposition and hotspot formation depends on nutrient availability in the rhizosphere. Geoderma.

[69] Jing J., Cong W.F., Bezemer T.M. (2022). Legacies at work: Plant-soil-microbiome interactions underpinning agricultural sustainability. Trends Plant Sci..

[70] Shi J., Wang X., Wang E. (2023). Mycorrhizal Symbiosis in Plant Growth and Stress Adaptation: From Genes to Ecosystems. Annu. Rev. Plant Biol..

[71] Shi Y.C., Zheng Y.J., Lin Y.C., Huang C.H., Shen T.L., Hsu Y.C., Lee B.H. (2024). Investigation of the Microbial Diversity in the Oryza sativa Cultivation Environment and Artificial Transplantation of Microorganisms to Improve Sustainable Mycobiota. J. Fungi.

[72] Tedersoo L., Bahram M., Zobel M. (2020). How mycorrhizal associations drive plant population and community biology. Science.

